# Comparative analysis of drought-responsive and -adaptive genes in Chinese wingnut (*Pterocarya stenoptera* C. DC)

**DOI:** 10.1186/s12864-021-07470-z

**Published:** 2021-03-04

**Authors:** Yong Li, Yu-Tao Si, Yan-Xia He, Jia-Xin Li

**Affiliations:** 1 Innovation Platform of Molecular Biology, College of Landcape and Art, Henan Agricultural University, Zhengzhou, China; 2 School of Life Sciences, Henan University, Kaifeng, China

**Keywords:** Drought-adaptive gene, Drought-responsive gene, Drought stress, *Pterocarya stenoptera*, Transcriptome

## Abstract

**Background:**

Drought is the main stress factor for the cultivation of *Pterocarya stenoptera* in urban areas, and this factor will cause its dehydration and affect its growth. Identifying drought-related genes will be useful for understanding the drought adaptation mechanism of *P. stenoptera*.

**Results:**

We used physiological indicator detection, comparative transcriptome sequencing, and reanalysis on the results of previous landscape genomics studies to investigate the drought adaptation mechanism in *P. stenoptera*. The changes in malondialdehyde content showed that *P. stenoptera* was remarkably affected by drought stress, and the increase in soluble sugar content suggested its important role in response to drought stress. Results of comparative transcriptome sequencing showed that *P. stenoptera* initiated a series of programs, such as increasing the gene expression of unsaturated fatty acids, tyrosine, and plant pathogen resistance, to deal with the transient drought stress. According to the annotated results in a previous study, *P. stenoptera* adapts to the long-term differential drought stress by regulating the thickness of cell walls and expressing upper or lower limits of the downstream genes in the hormone signaling pathway. Through the comparative analysis of drought-responsive and -adaptive genes in *P. stenoptera*, this study supports the hypothesis that the environment-responsive genes (ERGs) introduced by the transient environmental stresses will be substantially more than the environment-adaptive genes (EAGs) in response to long-term differential environmental stresses, and the EAGs are not necessarily ERGs.

**Conclusions:**

Our study identified drought-responsive and -adaptive genes in *P. stenoptera* and revealed that *P. stenoptera* increased the gene expression of unsaturated fatty acids, tyrosine, and plant pathogen resistance in response to transient drought stress. This study reveals the different adaptation mechanism of *P. stenoptera* under the transient and long-term differential drought stresses.

**Supplementary Information:**

The online version contains supplementary material available at 10.1186/s12864-021-07470-z.

## Background

The rapid global climate change is aggravating the environmental stresses faced by plants under the field, such as high temperature, cold, drought, and soil salinization [1, 2]. Among these environmental stresses, drought stress is an important factor that limits plant growth and development [3, 4]. Plants will undergo morphogenesis or physiological changes in response to drought stress [5, 6]. These morphogenesis or physiological changes are usually due to the changes in sequence and expression of drought-related genes. Identifying the drought-related genes will be useful for understanding the drought adaptation mechanism of *P. stenoptera* [7].

At present, two strategies are generally adopted to identify genes related to environmental stress at the genome level. The first strategy is the comparative transcriptome analysis by using the samples before and after environmental stresses. The related genes are up- or down-regulated under the stimulation of environmental stresses [8]. The species quickly respond to environmental stresses through the transient up- or down-regulation in gene expression. The genes identified by this strategy are defined as environment-responsive genes (ERGs) in this study. Increasing studies using this strategy improve the understanding of the adaptation mechanism of plants [9]. The second strategy is environment correlation analysis in landscape genomics. Individuals with different genotypes in the natural population have different survival or reproduction rates due to the spatial environmental heterogeneity [10, 11]. These environmental stresses leave selection signals on the genome of species after a long period of natural selection [12]. The genes related to environmental stresses are identified through gene detection with selection signals and correlation analysis between the genes and the corresponding environmental factors [13]. This strategy has been confirmed to be a highly effective approach in many previous studies [14–16]. The identified genes using the second strategy show sequence adaptive differentiation under the long-term natural selection, while those identified by the first strategy demonstrate changes in their expression under the short-term environmental stresses. The genes identified by the second strategy are defined as environment-adaptive genes (EAGs). However, limited studies have focused on the relationships between the ERGs and EAGs identified by the two strategies. Therefore, a new hypothesis is proposed as follows: the ERGs introduced by the transient environmental stresses will be considerably higher than the EAGs caused by the long-term differential environmental stresses, and the EAGs are not necessarily ERGs.

Chinese wingnut (*Pterocarya stenoptera* C. DC, Juglandaceae) is a dominant tree species in deciduous broad-leaved forests in China’s warm temperate and subtropical regions. This species grows along streams and in wetlands in the field. It is frequently used in urban landscaping in recent years because of its excellent ornamental properties [17]. When *P. stenoptera* is used as an urban landscaping tree, the water supply is obviously less than when grown in the field, this condition results in drought stress. Recent studies in *P. stenoptera* suggested that drought stress has remarkable effects on multiple physiological indicators, and long-term drought stress causes seedling death [18, 19]. To date, no published studies have reported the molecular adaptation mechanism in *P. stenoptera* under drought stress. The landscape genomics study on *P. stenoptera*, which provides insight into EAGs, has been recently conducted [20].

Transcriptomic analysis and physiological index detection were performed in this study to investigate the gene expression and physiological process changes in *P. stenoptera* under simulated drought stress. Comparative analysis between the drought-responsive genes (DRGs) and drought-adaptive genes (DAGs) identified in previous studies was conducted to reveal the relationships between them. The main objectives of the present study are as follows: (i) screen for drought-tolerant ERGs and characterize the adaptation mechanism in response to transient drought stresses, and (ii) reveal the relationships of ERGs introduced by the transient environmental stresses and the EAGs caused by the long-term differential environmental stresses.

## Results

### Physiological changes under simulated drought stress treatment

**Fig. 1:**
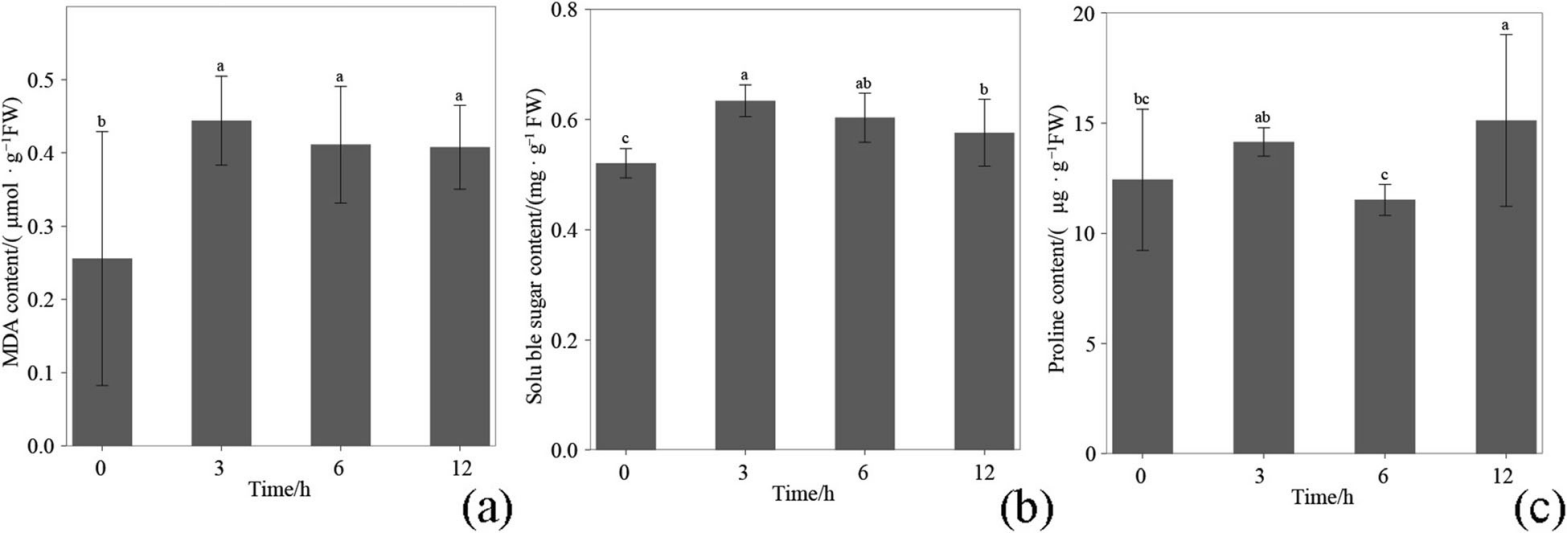
The content of **a** MDA, **b** SS, **c** Pro in leaves of *Pterocarya stenoptera* after 0 h, 3 h, 6 h, and 12 h exposure to drought stress conditions. The data represents mean values ± SD (*n* = 3). Different letters indicate significant difference at *P* < 0.05

The malondialdehyde (MDA) content in leaf under the simulated drought stress was obviously higher than that under the control, while that after cold stress did not continuously increase (Fig. 1a). The results showed that the membrane system was damaged by simulated drought stress. Moreover, soluble sugar (SS) substantially increased after simulated drought stress and then gradually decreased from 6 h to 12 h, but the SS contents at 6 and 12 h were still higher than those in the control (Fig. 1b). However, the proline (Pro) content did not demonstrate remarkable changes after simulated drought stress (Fig. 1c).

### Molecular responses under simulated drought stress

Clean reads of the nine cDNA libraries of *P. stenoptera* treated by control and drought at early (3 h) and late (12 h) stages ranged from 40,451,520 to 45,950,810, GC percentages ranged from 45.39 to 46.75%, and mapped reads ranged from 37,684,795 to 42,896,134 (Additional file 1: Table S1). The Q30 values of nine sequencing samples varied from 92.8 to 93.6%, which indicated that the output data were qualified for further analysis. The sequencing data were stored in the National Center for Biotechnology Information database (SRX7187821–7187829; BioProject PRJNA589251).

**Fig. 2:**
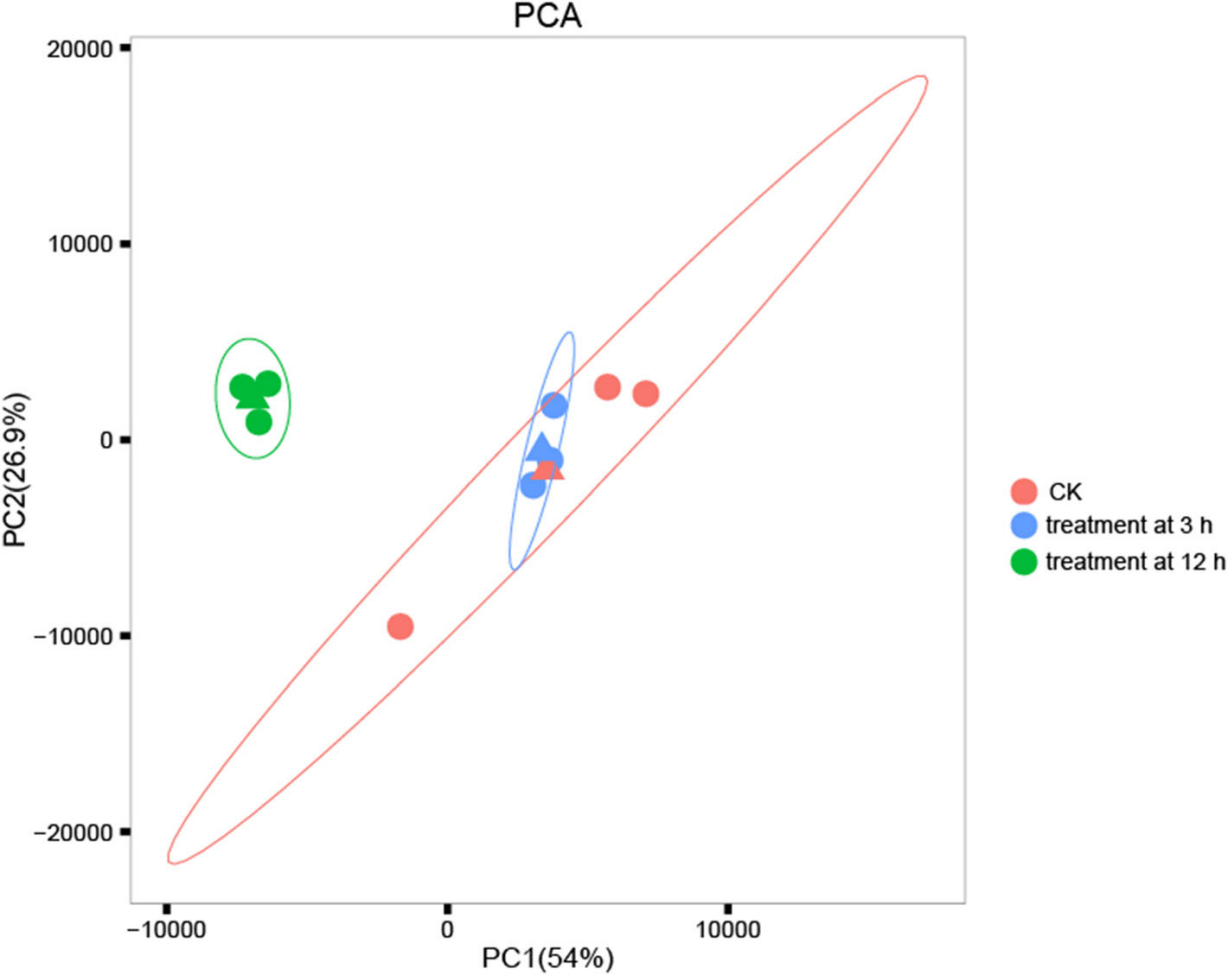
Principal component analysis of gene expression based on FPKM. Principal component 1 (PC1; 54% of variance) plotted against principal component 2 (PC2; 26.9% of variance). Red symbols correspond to control samples, blue symbols to treatment samples at 3 h, green symbols to treatment samples at 12 h

RNA-seq provided information on the DRGs in *P. stenoptera* under drought stress at early and late stages. Principal component analysis (PCA) was used to identify expression differences of all genes among all samples (Fig. 2). The PCA loading plot indicated that the effect of drought stress on the overall gene expression of the samples was small at 3 h, but it was significant at 12 h.

**Fig. 3:**
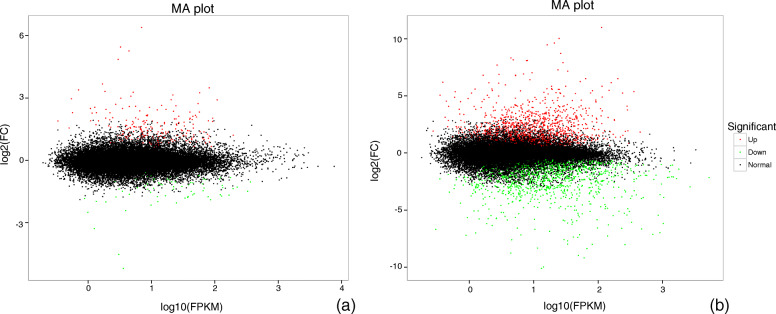
The MA plot to display the different expressed genes in leaves of *Pterocarya stenoptera* after 3 h (**a**) and 12 h (**b**) exposure to drought stress conditions when FC ≥ 1.5. The abscissa indicates the value of the Log2(FC) difference between the two groups; the ordinate indicates the value of the log10(FPKM) of the two groups. The green dots represents down-regulated genes, the red dots represents up-regulated genes, and the black dots represents non-differentially expressed genes

**Fig. 4:**
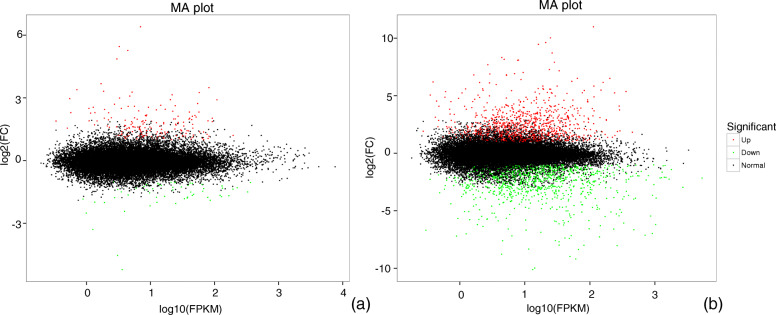
The MA plot to display the different expressed genes in leaves of *Pterocarya stenoptera* after 3 h (**a**) and 12 h (**b**) exposure to drought stress conditions when FC ≥ 2. The abscissa indicates the value of the Log2(FC) difference between the two groups; the ordinate indicates the value of the log10(FPKM) of the two groups. The green dots represents down-regulated genes, the red dots represents up-regulated genes, and the black dots represents non-differentially expressed genes

Compared with the control, 290 genes including 210 up-regulated and 80 down-regulated genes were significantly differently expressed at the early stage of drought stress when the parameter was set to fold-change (FC) ≥ 1.5 (Fig. 3a). Based on these DRGs, 5 pathways and 12 ontologies were up-regulated, 1 pathway and 5 ontologies were down-regulated (Table 1). With the extension of stress time, DRGs significantly increased, and 2374 genes including 1166 up-regulated and 1208 down-regulated genes were differentially expressed at the late stage (Fig. 3b). Based on these DRGs, 4 pathways and 3 ontologies were up-regulated, 5 pathways and 30 ontologies were down-regulated (Table 2). Compared with the control, 233 genes including 174 up-regulated and 59 down-regulated genes were significantly differently expressed at the early stage of drought stress when the parameter was set to FC ≥ 2 (Fig. 4a). Based on these DRGs, 1 pathway and 2 ontologies were up-regulated and 2 pathways and 8 ontologies were down-regulated (Additional file 1: Table S2). With the extension of stress time, DRGs significantly increased, and 2090 genes including 1044 up-regulated and 1046 down-regulated genes were differentially expressed at the late stage (Fig. 4b). Based on these DRGs, 6 pathways and 9 ontologies were up-regulated and 4 pathways and 25 ontologies were down-reguated (Additional file 1: Table S3).

**Table 1:** The GO and KEGG enrichment for up- and down- regulated DEGs at the early stage (3 h) with FC ≥ 1.5

ID	Description	*p*-value	*q*-value
**Up-regulated**			
**KEGG Enrichment**			
ko03008	Ribosome biogenesis in eukaryotes	1.46E-11	1.36E-09
ko04626	Plant-pathogen interaction	1.07E-08	9.91E-07
ko00071	Fatty acid degradation	8.46E-07	7.87E-05
ko00592	alpha-Linolenic acid metabolism	9.29E-07	8.64E-05
ko04712	Circadian rhythm - plant	1.53E-05	1.42E-03
**GO Enrichment**			
GO:0032549	ribonucleoside binding	1.23E-06	5.48E-04
GO:0003899	DNA-directed 5′-3′ RNA polymerase activity	3.15E-06	1.40E-03
GO:0009982	pseudouridine synthase activity	1.44E-05	6.41E-03
GO:0004004	ATP-dependent RNA helicase activity	1.79E-05	7.98E-03
GO:0005739	mitochondrion	3.59E-06	5.27E-04
GO:0009570	chloroplast stroma	1.43E-05	2.10E-03
GO:0032040	small-subunit processome	1.44E-05	2.12E-03
GO:0006351	transcription, DNA-templated	6.58E-09	3.83E-06
GO:0000373	Group II intron splicing	2.49E-07	1.45E-04
GO:0050789	regulation of biological process	7.51E-07	4.37E-04
GO:0010478	chlororespiration	5.22E-06	3.04E-03
GO:0010501	RNA secondary structure unwinding	8.14E-06	4.74E-03
**Down-regulated**			
**KEGG Enrichment**			
ko04141	Protein processing in endoplasmic reticulum	2.44E-05	1.54E-03
**GO Enrichment**			
GO:0004512	inositol-3-phosphate synthase activity	1.22E-08	1.95E-06
GO:0000155	phosphorelay sensor kinase activity	9.80E-06	1.57E-03
GO:0006021	inositol biosynthetic process	1.47E-08	3.37E-06
GO:0019419	sulfate reduction	7.26E-08	1.67E-05
GO:0009638	phototropism	2.92E-06	6.72E-04

**Table 2:** The GO and KEGG enrichment for up- and down- regulated DEGs at the late stage (12 h) with FC ≥ 1.5

ID	Description	*p*-value	*q*-value
**Up-regulated**			
**KEGG Enrichment**			
ko04712	Circadian rhythm - plant	8.46E-14	5.92E-12
ko00350	Tyrosine metabolism	4.24E-06	2.97E-04
ko00592	alpha-Linolenic acid metabolism	9.60E-06	6.72E-04
ko00591	Linoleic acid metabolism	1.18E-04	8.26E-03
**GO Enrichment**			
GO:0016168	chlorophyll binding	6.69E-04	3.02E-06
GO:0004022	alcohol dehydrogenase (NAD) activity	7.83E-04	3.54E-06
GO:0019887	protein kinase regulator activity	4.75E-03	2.15E-05
**Down-regulated**			
**KEGG Enrichment**			
ko00196	Photosynthesis - antenna proteins	4.94E-32	5.23E-30
ko00195	Photosynthesis	1.25E-13	1.33E-11
ko00710	Carbon fixation in photosynthetic organisms	6.04E-07	6.40E-05
ko01200	Carbon metabolism	1.36E-05	1.44E-03
ko00860	Porphyrin and chlorophyll metabolism	7.30E-05	7.74E-03
**GO Enrichment**			
GO:0016168	chlorophyll binding	2.72E-31	1.35E-28
GO:0031409	pigment binding	3.98E-18	1.97E-15
GO:0010277	chlorophyllide a oxygenase [overall] activity	4.74E-11	2.35E-08
GO:0030267	glyoxylate reductase (NADP) activity	2.50E-07	1.24E-04
GO:0016618	hydroxypyruvate reductase activity	2.50E-07	1.24E-04
GO:0016620	oxidoreductase activity, acting on the aldehyde or oxo group of donors, NAD or NADP as acceptor	4.69E-07	2.32E-04
GO:0009881	photoreceptor activity	5.83E-07	2.89E-04
GO:0051537	2 iron, 2 sulfur cluster binding	2.64E-06	1.31E-03
GO:0000155	phosphorelay sensor kinase activity	1.08E-05	5.35E-03
GO:0004512	inositol-3-phosphate synthase activity	1.13E-05	5.58E-03
GO:0015112	nitrate transmembrane transporter activity	1.35E-05	6.68E-03
GO:0008878	glucose-1-phosphate adenylyltransferase activity	1.35E-05	6.68E-03
GO:0009522	photosystem I	7.29E-32	9.69E-30
GO:0010287	plastoglobule	4.63E-27	6.15E-25
GO:0009535	chloroplast thylakoid membrane	2.10E-13	2.79E-11
GO:0009507	chloroplast	5.18E-08	6.90E-06
GO:0009654	photosystem II oxygen evolving complex	5.61E-08	7.46E-06
GO:0009538	photosystem I reaction center	7.27E-06	9.67E-04
GO:0018298	protein-chromophore linkage	6.27E-33	3.85E-30
GO:0009768	photosynthesis, light harvesting in photosystem I	9.65E-23	5.93E-20
GO:0009416	response to light stimulus	9.88E-14	6.07E-11
GO:0009765	photosynthesis, light harvesting	2.15E-11	1.32E-08
GO:0015979	photosynthesis	1.62E-09	9.94E-07
GO:0042549	photosystem II stabilization	3.37E-08	2.07E-05
GO:0019252	starch biosynthetic process	2.55E-06	1.56E-03
GO:0032957	inositol trisphosphate metabolic process	5.79E-06	3.56E-03
GO:0009585	red, far-red light phototransduction	7.88E-06	4.84E-03
GO:0006021	inositol biosynthetic process	1.05E-05	6.42E-03
GO:0010143	cutin biosynthetic process	1.21E-05	7.45E-03
GO:0000160	phosphorelay signal transduction system	1.49E-05	9.17E-03

Consistent with the FC threshold values with ≥1.5 and 2, the pathway of circadian rhythm - plant was significantly up-regulated at 3 h, and the ontology of chlororespiration was significantly down-regulated (Table 1 and Additional file 1: Table S2). When the FC threshold dropped to ≥1.5, the genes in metabolism of alpha-linolenic acid metabolism and plant-pathogen interaction were also significantly up-regulated. At the time of drought stress for 12 h, alpha-linolenic acid metabolism, linoleic acid metabolism, plant-pathogen interaction, circadian rhythm-plant, and tyrosine metabolism were significantly up-regulated; at this time, the genes of multiple pathways and ontologies related to photosynthesis were significantly down-regulated (Table 2 and Additional file 1: Table S3). In addition, the genes in nitrate transport and starch synthesis were significantly down-regulated.

### Candidate DAGs in response to long term drought natural selection

We reanalyzed previously published data on adaptive evolution to identify DAGs in response to long-term drought natural selection. According to the LFMM results in previous landscape genomics studies, 188 SNPs were strongly associated with principal component 1 (PC1) axis. PC1 represented precipitation in the driest seasons in previous studies. Among these SNPs, 24 and 95 SNPs were respectively annotated by the KEGG and GO databases (Additional file 1: Table S3). The SNPs annotated by KEGG database were further analyzed in detail. A total of 14 genes were regarded as candidate DAGs based on the annotated results of 24 SNPs. Among these candidate DAGs, only 3 genes including 2 up- and 1 down-regulated genes,were DRGs under the simulated drought stress treatment.

### Quantitative real-time PCR validation

Eight DRGs consisting of 5 up- and 3 down-regulated genes under drought stress were used to verify the RNA-Seq results through quantitative real-time PCR (*q*RT-PCR). The eight genes were mostly related to posttranslational modification, lipid and amino acid transport and metabolism, and energy production and conversion (Additional file 1: Table S4). The RNA-seq data was confirmed to be reliable based on the DRGs of *q*RT-PCR results. Pearson correlation coefficient (*r* = 0.756, *P* < 0.001) showed that significant positive correlation existed between *q*RT-PCR and RNA data.

## Discussion

Plants respond to drought stress through a series of morphogenesis, physiological, and molecular processes [21–23]. The effective high-throughput sequencing technique considerably facilitates the investigation of the adaptation mechanisms of drought stress [24]. Revealing the signaling pathways responsible for drought stress will provide clues for the cultivation and maintenance of urban landscaping plants.

*P. stenoptera* is widely used in landscape greening due to its excellent ornamental properties [17]. It is usually used in cities for street trees and solitary tree planting in parks. However, the water supply of *P. stenoptera* in urban cultivation is substantially lower than that in the field. The hardening of urban road surface reduced the infiltration of rain water and intensified the drought stress on *P. stenoptera*. Thus, *P. stenoptera* shows obvious dehydration symptoms in its leaves. For better cultivation and maintenance of *P. stenoptera*, it is necessary to detect the adaptive response of *P. stenoptera* under drought stress. Here, the physiological indicators and gene expression of *P. stenoptera* were determined. The increase in MDA content indicated that drought stress damaged the membrane system of *P. stenoptera*. In response to the change in cellular osmotic pressure introduced by the damage of the membrane system, the SS content significantly increased at the early stage of drought stress. The same response pattern has been found in other previous reports [1, 25]. However, the Pro content did not demonstrate significant changes compared with those in control. This finding indicated that Pro did not participate in the regulation of osmotic pressure under drought stress.

The results of RNA-seq showed that 290 and 2374 DRGs with FC ≥ 1.5 at the early and late stages were introduce by drought stress. The up- or down- regulated pathways and ontologies of DRGs showed a considerable amount of information on *P. stenoptera* response or adaptability under drought stress. At the early stage, the circadian rhythm-plant, alpha-linolenic acid metabolism, and plant-pathogen interaction were significantly up-regulated. The genes in circadian rhythm plays an important role in the response and adaptation of plants to environmental stress. Recent study showed drought impacts the oscillation of circadian clock genes in soybean [26]. Our results showed that drought significantly affected the endogenous rhythm system of *P. stenoptera*, which might induce a complex network regulation system, including the expression of DRGs. In plants, fatty acid metabolic pathways play a key role in plant defense. Low level of gene expression in alpha-linolenic acid can increase the damage degree on plants under drought stress. Increasing the synthesis of linolenic acid can reduce the effects of drought stress on plants [27]. Our results also showed that the drought tolerance of *P. stenoptera* was improved by increasing the gene expression of alpha-linolenic acid. The differential expression genes caused by drought stress and plant antigens have a crosstalk. Drought stress induces an overall response of key plant hormones that not only respond to water stress, but also play a key role in plant responses to pathogens [28]. The genes related to plant-pathogen interaction pathway are usually up-regulated under drought stress [29]. Over all, the genes of pathways of circadian rhythm - plant, alpha-linolenic acid metabolism and plant-pathogen interaction were up-regulated to respond to drought stress in the early stage of *P. stenoptera*. At the late stage, most down-regulated pathways and ontologies are associated with chloroplast and photosynthesis, which showed that photosynthesis is significantly affected by drought stress. This significant effect of drought stress on photosynthesis has also been reported in other studies [30, 31]. In addition, the down-regulated DRGs related to nitrate transport and starch synthesis showed that drought stress might limit the nutrient absorption and carbohydrate synthesis of plants [32]. Drought stress affected the growth of *P. stenoptera* and decreased the expression of genes in photosynthetic and nutrient absorption. In the late stage, several metabolic pathways, namely, circadian rhythm-plant, alpha-linolenic acid metabolism, and plant-pathogen interaction,that were upregulated in the early stage were found. In addition, the up-regulated genes were increased in linoleic acid metabolism and tyrosine metabolism. Linoleic acid metabolism and alpha-linolenic acid metabolism share a common pathway, and multiple genes overlap between them. The DRGs related to alpha-linolenic acid metabolism and linoleic acid metabolism suggested that *P. stenoptera* increased drought tolerance by maintaining membrane fluidity and integrity by modulating linolenic acid levels during drought stress [33]. The DRGs related to tyrosine enhance stress resilience by influencing osmotic changes and ROS detoxification [34].

Overall, the DRGs provided a considerable amount of information on the response of *P. stenoptera* to the transient drought stress. Under the transient drought stress, *P. stenoptera* initiated a series of programs, including increasing the gene expression of unsaturated fatty acids, tyrosine, and plant pathogen resistance, to cope with drought stress. Meanwhile, drought stress limited the nutrient absorption and carbohydrate synthesis in *P. stenoptera*.

Compared with a large amount of DRGs introduced by transient drought stress, a small amount of DAGs was produced in the natural population under the long-term differential drought stress. A total of 14 candidate DAGs were obtained on the basis of the annotated results from the SNPs identified by LFMM analysis using KEGG database [20]. The most identified DAGs did not overlap with DRGs, and only three DAGs were DRGs. Among these DAGs, caffeic acid 3-O-methyltransferase and cellulose synthase A enhanced drought tolerance by participating in cell wall synthesis [35, 36]. Ethylene-responsive transcription factor and molybdenum cofactor sulfurase increase drought tolerance by participating in the hormone signaling pathway [37, 38]. Overall, the number of DAGs is much smaller than that of DRGs. Although the two research strategies are used to search for drought related genes, they obviously find two types of genes in different meanings. In the dry season, natural populations suffer drought stress, while the degree of drought stress is different due to the spatial environmental heterogeneity. The population needs only a small number of gene sequence differentiation to treat this different intensity selection pressure. DRGs in these populations still play the most important role in the face of drought stress [39]. However, the DAGs of sequence differentiation can respond to different intensity drought stresses because of their different gene efficacies. The results of DAGs suggested that *P. stenoptera* adapted to the differential drought stress by regulating the thickness of cell walls and the upper or lower limits of the downstream genes in the hormone signaling pathway, while the results of DRGs suggested that *P. stenoptera* adapted to transient drought stress by increasing the gene expression of unsaturated fatty acids, tyrosine, and plant pathogen resistance. The abovementioned results indicate that DAGs identified by landscape genomics and DRGs identified by comparative transcriptomics have obviously different meanings. However, they are all related to drought, while DRGs will be significantly more than DAGs. Our results also confirm that most of the two types of genes have only a small intersection. Although EAGs were considerably less than ERGs, these individuals with highly efficient EAGs were substantially useful in screening germplasm resources from natural populations for breeding. Overall, the present results support the hypothesis that the ERGs introduced by the transient environmental stresses will be considerably higher than the EAGs in response to long-term differential environmental stresses, and the EAGs are not necessarily ERGs.

## Conclusions

Physiological indicator detection, transcriptome sequencing, and reanalysis of the results of previous landscape genomics study were used to reveal the drought adaptation mechanism in *P. stenoptera*. The obtained results indicated that *P. stenoptera* increased the gene expression of unsaturated fatty acids, tyrosine and plant pathogen resistance, to respond to transient drought stress. *P. stenoptera* adapted to the long-term differential drought stress by regulating the thickness of cell walls and the upper or lower limits of the downstream genes in the hormone signaling pathway. The obtained results supported the hypothesis that the ERGs introduced by the transient environmental stresses will be considerably higher than the EAGs in response to long-term differential environmental stresses, and the EAGs were not necessarily ERGs. This study reveals the different adaptation mechanism of *P. stenoptera* under the transient and long-term differential drought stresses.

## Methods

### Culture and growth of plant materials

The *P. stenoptera* seeds were obtained from Shuyang Jiuluolika Seed Co., Ltd. in Jiangshu. Stratification treatment were firstly performed on these seeds under 4 °C for 30 days, the seeds were then sterilized in 0.5% K_2_M_n_O_4_ solution. Afterward, the seeds were transferred into culture dishes with two layers of wet filter paper until the radicle burst the testa. The seedlings were further replanted to a nutrient bowl (9 cm × 11 cm) with one strain per pot. The seedlings were cultured in the artificial climate chest (PLD-500-G4, Ledian Instrument Manufacturing Co., Ltd). The culture conditions were set as follows: 20,000 lx illumination intensity, 27/22 °C (14/10 h at day/night), and 60% air relative humidity. After the seedlings were cultured for 6 months, the whole seedlings were transplanted into a 100 mL conical flask containing 1/2 Hoagland’s nutrient solution for seedling recovery. The treatment of seedling recovery was conducted in the artificial climate chest for 1 month. The seedlings with the same and vigorous growth were selected as the experimental materials. These seedlings were then treated by using the 1/2 Hoagland’s nutrient solution containing 10% (g mL^− 1^) polyethylene glycol (PEG-6000). The seedlings were treated at 0, 3, 6, and 12 h. The leaves of treated plants were randomly selected and immediately transferred into liquid nitrogen and finally stored in a refrigerator at − 80 °C. The experimental treatments were designed with three replicates to ensure data reproducibility. The voucher specimen of *P. stenoptera* were identified by Dr. Yong Li and deposited at the herbarium of College of Landscape and Art, Henan Agricultural University (voucher no. LiPS2019A01).

### Detection of physiological indicators

Three physiological indicators, that is, the contents of MDA, SS, and Pro, were determined to reflect the effects of drought on the *P. stenoptera* seedlings. MDA is the product of membrane lipid peroxidation, which reflects the damage degree of membrane system under drought stress [40]. The MDA content was determined using thiobarbituric acid method [41]. SS and Pro are both important osmoregulation substances, which can reduce the cell osmotic potential and enhance the water absorption capacity to improve the tolerance or adaptability of plants to drought stress [42]. The SS and Pro contents were determined according to the method of Rosa et al. [43] and Bates et al. [44], respectively. The three physiological indexes were detected using microplate reader Infinite M PLEX (Tecan, Grödig, Austria). These indexes were measured on 0, 3, 6, and 12 h after drought treatment. Three biological replications were set for each treatment. Significance of the results of the physiological indexes were determined using the least-significant difference test (*P* < 0.05) in one-way analysis of variance (ANOVA).

### Library construction and transcriptome sequencing

RNA analyses were performed at 3 and 12 h after drought treatment, the samples before drought treatment were set as control. All treatments had three bioligical replications. Total RNA was isolated using a plant total RNA Extraction Kit DP432 (Tiangen Technologies, Beijing, China). RNA quality was evaluated using Agilent 2100 (Agilent Technologies, CA, USA) and NanoDrop 2000 (Thermo Fisher Scientific, DE, USA). The sequencing libraries were constructed by using NEBNext Ultra™ RNA Library Prep Kit according to the manufacturer’s protocols. The library quality was further evaluated using Agilent 2100 (Agilent Technologies, CA, USA). Finally, the qualified libraries were sequenced on the HiSeq X Ten system (BioMarker Technologies, Beijing, China).

### Mapping sequence reads to the reference genome

After removing the adapter sequences and low-quality reads from raw reads, the remaining clean reads were subsequently mapped on the draft genome of *P. stenoptera* [45] by using the software HISAT2 [46] and further assembled by using the software StringTie [47].

### Identification and functional annotation of differentially expressed genes (DEGs)

The gene expression levels in the samples were measured by fragments per kilobase of exon per million fragments mapped using the software StringTie [47]. The average value of FPKM of three samples for each treatment was taken when DEGs was identified. PCA was further performed based on FPKM value of all expressed genes using the plotPCA function in DESeq2 [48] to reveal the broad patterns of variation of nine samples in response to drought stress. The DEGs in *P. stenoptera* introduced by drought stress were obtained using DESeq2 software [48]. The selection criteria for DEGs was set as false discovery rate < 0.01 and FC ≥ 1.5 and 2. The function of the DEGs was annotated using the KEGG [49] and GO [50] databases. The identified DEGs were redefined as DRGs due to their rapid response to drought stress. To identify the enriched GO ontologies and KEGG pathways of these DEGs, the statistical enrichment analyses were implemented using the topGO packages [51] in R and KOBAS [52]. The significant of enrichment pathways and ontologies were determined using *q*-values < 0.01 as criteria.

### Reanalysis of the results of landscape genomics

To search candidate DAGs, we reanalyzed previously published data [20]. According to previous landscape genomics study, precipitations of the driest month (Bio14) and quarter (Bio17) were strongly associated with PC1 axis [20]. Therefore, the genes related to PC1 axis can be regarded as the candidate DAGs. The identified DAGs from the LFMM analysis [53] were reannotated and classified in this study using the KEGG and GO databases.

### *q*RT-PCR validation

Eight DRGs with the corresponding primers (Addtional file 1: Table S4) were selected to validated their expression levels using *q*RT-PCR. The *q*RT-PCR reactions were performed with the TB Green Premix Ex Taq II (TaKaRa, China) and carried on the ABI QuantStudio®3 Real-Time System (Applied Biosystems, USA). The PCR conditions were an initial denaturation at 95 °C for 10 min, 40 cycles of 95 °C for 15 s, and 60 °C for 1 min. The 18S rRNA using in *Juglans regia* [54] was selected as an internal reference gene, and all the reactions were repeated three times. The expression levels of eight DRGs were caculated by using the relative 2^−△△Ct^ method [55].

## Supplementary Information

**Additional file 1.** The supporting data for this manuscript. **Table S1.** Summary of sequence data from nine samples. **Table S2.** The GO and KEGG enrichment for up- and down- regulated DEGs at the early stage (3 h) with FC ≥ 2. **Table S3.** The GO and KEGG enrichment for up- and down- regulated DEGs at the late stage (12 h) with FC ≥ 2. **Table S4.** The primers of qRT-PCR and gene function for 8 selected genes. (XLS 44 kb)

## Data Availability

The datasets generated and analysed during the current study are available in the National Center for Biotechnology Information repository, SRX7187821–7187829, BioProject PRJNA589251. The draft genome of *P. stenoptera* are available in https://www.ncbi.nlm.nih.gov/assembly/GCA_003123785.1/.

## Abbreviations

Bio14: precipitations of the driest month
Bio17: precipitations of the driest quarter
DAGs: drought adaptive genes
DEGs: differentially expressed genes
DRGs: drought responsive genes
EAGs: environment adaptive genes
ERGs: environment responsive genes
FC: fold-change
GO: Gene Ontology
KEGG: Kyoto Encyclopedia of Gene and Genome
MDA: malondialdehyde
PC1: principal component 1
Pro: proline
ROS: reactive oxygen species
SS: soluble sugar
